# SAHA Alters Macrophages in the Tumor-Immune Landscape in Preclinical Models of Triple-Negative Breast Cancer

**DOI:** 10.3390/pharmaceutics18050539

**Published:** 2026-04-28

**Authors:** Shannon E. Lynch, Corinne I. Crawford, Troy D. Randall, Patrick N. Song, Renata Jaskula-Sztul, Anna G. Sorace

**Affiliations:** 1 Graduate Biomedical Sciences, University of Alabama at Birmingham, Birmingham, AL 35233, USA; slynch97@uab.edu (S.E.L.);; 2 Department of Radiology, University of Alabama at Birmingham, Birmingham, AL 35233, USA; 3 Department of Biomedical Engineering, University of Alabama at Birmingham, Birmingham, AL 35233, USA; 4 Department of Immunology and Rheumatology, University of Alabama at Birmingham, Birmingham, AL 35233, USA; 5 O’Neal Comprehensive Cancer Center, University of Alabama at Birmingham, Birmingham, AL 35233, USA; 6 Department of Surgery, University of Alabama at Birmingham, Birmingham, AL 35233, USA

**Keywords:** triple-negative breast cancer, HDAC, immunotherapy, SSTR2, macrophages

## Abstract

**Background/Objectives**: Histone deacetylase (HDAC) inhibitors have been shown to prime the response to immunotherapy (IMT) treatment by inducing immune activation and infiltration to target tumor cells. Many studies primarily focus on adaptive immune cells and their expression of pro-inflammatory markers, like somatostatin receptor 2 (SSTR2); however, macrophages are known to help mediate key tumor microenvironment changes. The goal of this study is to evaluate the effects of HDAC inhibitors and IMT on macrophages, their expression of SSTR2, and their impact on the treatment response in triple-negative breast cancer (TNBC). **Methods**: Cytotoxic effects of HDAC inhibitors on 4T1 mouse mammary carcinoma cells, including suberoylanilide hydroxamic acid (SAHA), were evaluated using flow cytometry. Bone marrow-derived macrophages (BMDMs) were stimulated to M1-like and M2-like phenotypes and treated with SAHA to explore the effects on SSTR2 expression in different macrophage phenotypes. 4T1-tumor-bearing BALB/c mice were used to evaluate the therapy response to four treatments: saline control, SAHA, anti-PD-1 + anti-CTLA-4 checkpoint blockade IMT, or a combination of SAHA + IMT. Additional cohorts of 4T1-tumor-bearing BALB/c mice and NOD SCID mice, which lack adaptive immune cells, were euthanized for early evaluation of tumor-associated macrophage (TAM) populations via flow cytometry and cytokine analysis. One-way independent ANOVAs and log-rank tests were used to compare group differences. **Results**: SAHA promotes SSTR2 expression on M1-like BMDMs in vitro. SAHA promotes M2-like TAMs in vivo and stimulates pro-inflammatory, anti-tumor cytokine production in combination with IMT. **Conclusions**: SAHA drives SSTR2 expression and anti-tumor innate immune responses with additive effects in combination with immunotherapy in preclinical TNBC.

## 1. Introduction

Histone deacetylase (HDAC) inhibitors are a class of drugs that prevent the removal of acetyl groups from histones, leaving chromatin open for transcription and ultimately activating downstream signaling cascades [1,2]. These epigenetic changes are responsible for the regulation of transcription factors, cell signaling, and protein synthesis and degradation, which can influence different physiological pathways ranging from tumor progression to T cell activation [1]. Importantly, pan-HDAC inhibitors are currently being explored in breast cancer in combination with chemotherapy (NCT00616967, NCT00574587, NCT01084057), PARP inhibitors (NCT03742245), and immunotherapy (NCT04190056, NCT02395627) with potential for additive or synergistic effects. Triple-negative breast cancer (TNBC) is highly heterogeneous and lacks effective treatment options due to the absence of molecular targets, leading to high metastatic potential and poor prognosis clinically when compared to other breast cancer subtypes [3,4,5]. While approved treatments, including surgery, chemotherapy, radiation, and immunotherapy (IMT), significantly improve patient survival, they are only effective in about 20% of patients [6]. Therefore, novel combination therapies, such as adding HDAC to IMT, provide a potential avenue to improve the effectiveness of currently available treatment options.

There has been increased interest in the role of HDAC inhibitors to enhance the tumor-immune microenvironment, which plays an important role in mediating cancer cell apoptosis, lymphocyte recruitment and infiltration, and the overall cancer treatment response. HDAC inhibitors have been shown to promote the production of inflammatory cytokines and enhance T cell activation in tumors to induce apoptosis of cancer cells, but the specific mechanism is not well understood [7,8,9,10,11]. Studies in other cancer models, most notably melanoma, have shown that treatment with the HDAC inhibitor Vorinostat (suberoylanilide hydroxamic acid (SAHA)) can prime immune responses and have additive benefits to IMT treatment [12,13]. Some studies attribute the effect of increased cytotoxic T cell activation and killing of tumor cells to upregulation of major histocompatibility class I and II (MHC Class I and II) expression on tumor cells downstream of HDAC inhibition [14,15,16]. Other notable studies attribute increased promotion and production of pro-inflammatory cytokines, like IL-6, IL-8, IL-1β, TNFα, and IFNγ, in the tumor microenvironment (TME), which further stimulates pro-inflammatory cells like M1 macrophages and induces cytotoxic T-lymphocyte-mediated cell death [17,18]. Though macrophages play key roles in tumor progression and lymphocyte recruitment and activation, the effects of combination therapies on these cells remain relatively understudied.

Macrophages mediate key hallmarks of cancer including inflammation, angiogenesis, and metastasis through the secretion of cytokines and growth factors. Tumor-associated macrophages (TAMs) in the TME influence processes important to cancer growth and metastasis including antigen presentation, remodeling of the extracellular matrix, and recruitment of lymphocytes [19,20]. Macrophages have a wide variety of complex functions but can typically be classified as either M1-like, which are pro-inflammatory and anti-tumoral, or pro-wound healing and pro-tumoral, referred to as M2-like [21]. The current literature suggests that the G-protein-coupled receptor somatostatin receptor 2 (SSTR2) is expressed on some human monocytes and macrophages and has a pro-inflammatory effect [4,18,22,23]. Macrophages and other lymphocytes, like T cells and natural killer cells, communicate with one another via enzymes and cytokines to recruit other lymphocytes and induce apoptosis of cancer cells. Though macrophages mediate key hallmarks of cancer, there are few studies which focus on the effects of HDAC inhibitors and IMT on macrophages. Investigating how the tumor-immune microenvironment is influenced by the addition of HDAC inhibitors to immunotherapy could impact the treatment response in TNBC.

Though it has been established that SSTR2 is expressed on macrophages, understanding how SAHA influences this expression remains unexplored. Previous data from our group have shown that SAHA treatment increases SSTR2 expression at the transcriptional, translational, and functional levels on tumor cells in two preclinical models of TNBC [22]. Due to the indirect action of HDACi and direct influences of IMT on macrophages, these studies evaluate if the combination of HDACi—specifically, SAHA—and immunotherapy increases SSTR2 expression and recruits pro-inflammatory macrophage phenotypes. The goal of these studies is to determine whether SAHA upregulates SSTR2 on M1-like macrophages and understand how these cells influence immunotherapy response in TNBC. We aim to evaluate the effects of combination therapy on macrophages directly and indirectly to better understand how they influence treatment response.

## 2. Materials and Methods

### 2.1. Cancer Cell Culture

4T1 TNBC mouse mammary carcinoma cells were purchased from American Type Culture Collection (ATCC, Manassas, VA, USA). Cells were cultured in RPMI 1640 medium (Thermo Fisher Scientific, Waltham, MA, USA) supplemented with 10% (*v*/*v*) fetal bovine serum (FBS, Biotechne S12450H R&D Systems, Denver, CO, USA), 1% (*v*/*v*) penicillin/streptomycin, 1% (*v*/*v*) sodium pyruvate, and 1% (*v*/*v*) L-glutamine and grown at 37 °C with 5% CO_2_. Cells were cultured to 70–80% confluency, and cell viability was determined using trypan blue dye exclusion (Thermo Fisher Scientific, Waltham, MA, USA).

### 2.2. Animal Models

The study was conducted in accordance with UAB’s Institutional Animal Care and Use Committee (IACUC) guidelines and regulations, and the reporting of experiments is compliant with ARRIVE guidelines. Female mice only were used in these studies, as breast cancer is a predominantly sex-specific disease. Female mice (5–6 weeks of age) were housed N = 5/cage with ad libitum access to food and water on a standard 12 h light/dark cycle and acclimatized for 7 days prior to any experimentation. Researchers were not blinded to the treatment groups, as tumor volume changes due to treatment were recorded throughout. Procedures using animals, including housing, tumor inoculation, treatment administration, anesthesia via isoflurane, euthanasia via primary over-administration of 5% isoflurane and secondary cervical dislocation, and collection of biological materials, are approved under animal protocol number (APN) 08778.

#### 2.2.1. Tumor-Associated Macrophage Evaluation

**Figure 1:**
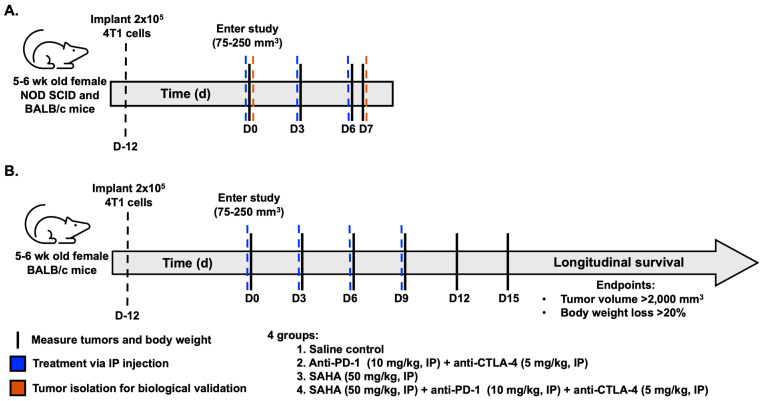
Experimental timeline for biological validation and therapy study. (**A**) Evaluation of tumor-associated macrophages (TAMs) in immunocompetent BALB/c (N = 12) and immunocompromised NOD SCID (N = 12) 4T1-tumor-bearing mice (N = 3/treatment group per model). (**B**) Longitudinal survival for BALB/c 4T1-tumor-bearing mice. Treatment groups for both studies include (1) Saline control, (2) SAHA, (3) IMT (anti-PD-1 and anti-CTLA-4), and (4) SAHA + IMT. Solid black line represents measurements of tumors via calipers and body weight every three days. Blue dotted lines represent treatment administration days. Orange lines represent tumor isolation at pre-determined endpoints for flow cytometry assessment of TAMs.

A total of 2 × 10^5^ 4T1 cells in saline were implanted into the third mammary fat pad of 5–6-week-old female BALB/c (N = 12, Figure 1A) or NOD SCID (N = 12, Jackson Labs #001303 Bar Harbor, ME, USA) mice. To understand the effects of SAHA and IMT on macrophages, NOD SCID animals were chosen to compare the effects of therapies in the absence of fully functional adaptive immune cells, primarily T and B cells, and compared to BALB/c mice with an intact immune system. Beginning on day 0, mice began one of four treatments: saline control, single-agent suberoylanilide hydroxamic acid (SAHA, SML0061 Sigma Aldrich, St. Louis, MO, USA), single-agent checkpoint blockade immunotherapy (α-PD-1 #BE0146 and α-CTLA-4 #BE0131 BioXCell, Lebanon, NH, USA), or a combination of SAHA and IMT. Therapies were administered every 3 days via intraperitoneal injection, and each animal received 4 total doses of each treatment. Tumor volume measurements via calipers and body weights were taken every 3 days. 4T1-tumor-bearing mice entered the study upon reaching a tumor volume of 75–250 mm^3^ (12 days after implantation), referred to as day 0. All NOD SCID (N = 3 per group) and a subset of BALB/c mice (N = 3 per group) were euthanized at day 7 of treatment as a pre-determined time point of interest for biological validation.

#### 2.2.2. SAHA/IMT Therapy Study

The remaining 4T1-tumor-bearing BALB/c animals were randomized into treatment groups using a random number generator (N = 28 total, N = 7 per group, Figure 1B), with quality checks to ensure equal tumor volume distributions between groups at baseline and monitoring for long-term survival. Tumor volume measurements via calipers and body weights were taken every 3 days. Tumor-bearing mice entered the study upon reaching a tumor volume of 75–250 mm^3^ (12 days after implantation), referred to as day 0. Beginning on day 0, mice began one of four treatments: saline control, single-agent suberoylanilide hydroxamic acid (SAHA, SML0061 Sigma Aldrich, St. Louis, MO, USA), single-agent checkpoint blockade immunotherapy (α-PD-1 #BE0146 and α-CTLA-4 #BE0131 BioXCell, Lebanon, NH, USA), or a combination of SAHA and IMT. Therapies were administered every 3 days via intraperitoneal injection, and each animal received 4 total doses of each treatment. SAHA was reconstituted in 30% polyethylene glycol 300 (*v*/*v*), 5% Tween 80 (*v*/*v*), 10% dimethylsulfoxide (DMSO, (*v*/*v*)), and 55% saline, per the manufacturer’s recommendation, and administered at 50 mg/kg based on previously established cytotoxicity (CC_50_) concentrations [22]. Endpoints for tumor-bearing animals include sustained tumor volume of 2000 mm^3^ or greater than 20% body weight loss for the longitudinal survival study.

### 2.3. Flow Cytometry

#### 2.3.1. Quantifying the Effects of SAHA on Cancer Cell Viability

**Figure 2:**
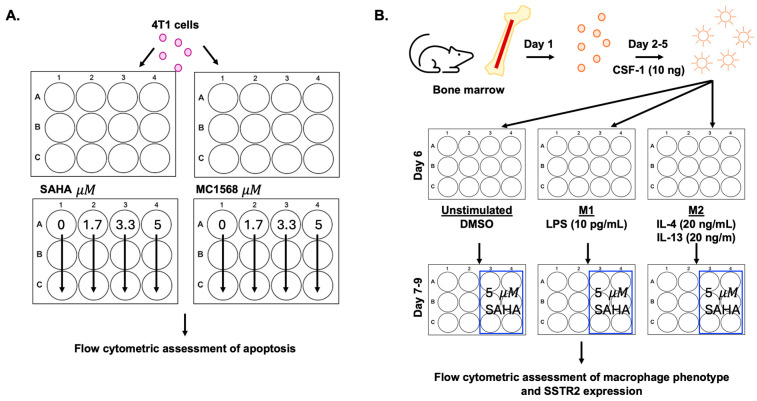
Experimental design to evaluate SAHA cytotoxicity on cancer cells and effect on polarization and SSTR2 expression for BMDMs. (**A**) 4T1 cancer cells were plated in 12-well plate overnight and then treated with increasing doses of HDAC inhibitors. Following 24 h of treatment, cells were assessed by flow cytometry for apoptosis using AF488-Annexin V and propidium iodide fluorescent dye. (**B**) Bone marrow-derived macrophage (BMDM) isolation, stimulation, and SAHA treatment timeline. Bone marrow was harvested and stimulated to differentiate macrophages using colony stimulating factor 1 (CSF-1). Following differentiation, BMDMs are stimulated to M1-like or M2-like phenotypes using cytokines, treated with SAHA, and assessed for phenotyping and SSTR2 expression by flow cytometry.

A total of 1 × 10^5^ 4T1 cells were plated in a 12-well plate and allowed to adhere overnight. 4T1 cells were then treated with increasing concentrations (0, 1.7, 3.3, and 5 µM; N = 3 biological replicates per treatment dose; Figure 2A) of class I and class II inhibitor SAHA or class II inhibitor MC1568 for 24 h. Following 24 h, cells were isolated using trypsin, and each sample was resuspended in 1× fluorescent-associated cell sorting (FACS, 1× PBS with 1% bovine serum albumin (*w*/*v*) and 0.1%. sodium azide (*w*/*v*)) buffer at a concentration of 1 × 10^6^ per mL. Cell staining to evaluate apoptosis was performed using a fluorophore-conjugate for phosphatidyl serine (PS)-binding Annexin V and the fluorescent DNA-binding dye propidium iodide (PI), which were both incubated at 4 °C for 30 min in the dark, and antibodies and dilutions can be found in Supplemental Table S1. Annexin V binds to PS within the inner cell membrane, which flips to the outer membrane when cells are damaged [23]. When the cell membrane becomes compromised, PI can permeabilize into the cell freely and bind double-stranded DNA, indicating cell death [24]. Healthy cells with intact cell membranes stain negatively for both annexin V and PI. Apoptotic cells were determined using annexin V+ and PI−, where necrotic cells were annexin V + PI+ [25]. Layouts for the gating strategy can be found in Supplemental Figure S1. Data was acquired using a 6-violet laser Attune N × T flow cytometer (Thermo Fisher, Waltham, MA, USA), and subsequent analysis was performed with FlowJo version 10.6.2 software (Becton, Dickinson and Company, Ashland, OR, USA).

#### 2.3.2. Assessing SSTR2 Expression on Bone Marrow-Derived Macrophages

##### Generation of Bone Marrow-Derived Macrophages (BMDMs)

Bone marrow was harvested by flushing the cavities of the femur and tibia from N = 4 female BALB/c mice with a 26-gauge needle containing DMEM medium (Thermo Fisher Scientific, Waltham, MA, USA) and then filtered over a 70 µM cell strainer and centrifuged at 1300 rpm for 5 min at room temperature (RT). Cell pellets were resuspended in 1× ACK lysis buffer (eBioscience 00-4300-54 Thermo Fisher Scientific, Waltham, MA, USA) at room temperature for 5 min to lyse red blood cells, then neutralized using DMEM growth medium. Cell viability was assessed via the Trypan blue exclusion method, and then cells were resuspended in DMEM medium supplemented with 10% (*v*/*v*) fetal bovine serum (FBS, Biotechne S12450H R&D Systems, Denver, CO, USA), 1% (*v*/*v*) penicillin/streptomycin, 1% (*v*/*v*) sodium pyruvate, 1% (*v*/*v*) L-glutamine, 0.005% β-mercaptoethanol (*v*/*v*), and 10 ng recombinant mouse macrophage colony stimulating factor 1 ((*w*/*v*) CSF-1, Gibco PMC2044 Thermo Fisher Scientific, Waltham, MA, USA), referred to as Mø media. Cells were plated at a concentration of 10 × 10^6^ per 100 mm dish and grown at 37 °C with 5% CO_2_. The following day, media containing non-differentiated cells were collected and centrifuged at 1300 rpm for 5 min at RT; cells were then resuspended and replated at a concentration of 10 × 10^6^ per 100 mm dish and grown at 37 °C with 5% CO_2_ (Figure 2B).

##### BMDM Stimulation

On day 6 post isolation, macrophages were dissociated from culture using 5 mM EDTA and neutralized using 1× PBS. Cells were resuspended in Mø media and plated at a concentration of 3 × 10^5^ per well in a 12-well plate for stimulation, as shown in Figure 2B (1 plate per stimulation group, 3 groups). Three separate plates were used for each stimulation condition including (1) unstimulated, (2) M1-like stimulation, or (3) M2-like stimulation. On day 7, media was changed to serum-free DMEM 2 h prior to stimulation. Two hours later, media was changed to stimulate macrophage polarization to the M1 phenotype (Mø media containing 10 pg/mL lipopolysaccharides from *E. coli* (O111:B4 Sigma Aldrich, St. Louis, MO, USA)) or the M2 phenotype (Mø media containing 20 ng/mL IL-4 (R&D 404-ML-010 Thermo Fisher Scientific, Waltham, MA, USA) and 20 ng/mL IL-13 (R&D 413-ML-005 Thermo Fisher Scientific, Waltham, MA, USA)).

##### SAHA Treatment of Stimulated BMDMs

At 18 h post M1 stimulation and 40 h post M2 stimulation, a subset of stimulated macrophages received treatment with 5 µM SAHA or dimethylsulfoxide (DMSO) as a control (N = 6 per group, Figure 2B). M1-stimulated macrophages were isolated from culture at 26 h post stimulation, and M2-stimulated macrophages were isolated from culture at 48 h post stimulation using 5 mM EDTA for analysis via flow cytometry. Layouts for the gating strategy can be found in Supplemental Figure S2. Antibodies and dilutions for cell staining of viability and macrophage populations can be found in Supplemental Table S1. Data was acquired and analyzed as previously described.

#### 2.3.3. Quantifying the Effects of SAHA Alone and in Combination with IMT on TAMs

Tumors were excised, cut into 1–2 mm pieces, and enzymatically digested using Type IV collagenase (Gibco 17104019 Thermo Fisher Scientific, Waltham, MA, USA) at 37 °C for 30 min followed by mechanical dissociation with a tissue homogenizer. Following digestion, red blood cells were lysed using 1× ACK lysis buffer (eBioscience 00-4300-54 Thermo Fisher Scientific, Waltham, MA, USA) at room temperature for 5 min then neutralized using RPMI growth medium. Remaining cells were then twice filtered through a 40 µm cell strainer, and cell viability was assessed via the Trypan blue exclusion method. Each sample was resuspended in 1× FACS buffer at a concentration of 1 × 10^6^ per mL. Non-specific binding was inhibited using Fc block (BD 553142 Becton, Dickinson and Company, Ashland, OR, USA), which incubated at 4 °C for 10 min prior to cell staining. Cell staining was performed using fluorophore-conjugates incubated at 4 °C for 30 min in the dark. Layouts for the gating strategy can be found in Supplemental Figure S2. Antibodies and dilutions for cell staining of viability and macrophage populations can be found in Supplemental Table S1. Data was acquired and analyzed as previously described.

### 2.4. Cytokine Analysis

Tumor samples were enzymatically digested using a collagenase solution and mechanically digested with a tissue homogenizer, as previously described in Section 2.3.3. Following filtration into a single-cell suspension, cells were centrifuged, and supernatants were isolated for the detection of chemokines and cytokines including CCL1, CCL11, CCL12, CCL17, CCL19, CCL2, CCL20, CCL22, CCL24, CCL3, CCL4, CCL5, CCL7, CX3CL1, CXCL1, CXCL10, CXCL11, CXCL12, CXCL13, CXCL16, CXCL2, CXCL5, GM-CSF, IFN-γ, IL-10, IL-16, IL-1β, IL-2, IL-4, IL-6, and TNF-α, which were determined using the Bio-Plex Pro Mouse Chemokine Panel 31-Plex kit (#12009159 BioRad, Hercules, CA, USA). Protein concentration was determined using a Pierce Rapid Gold BCA Protein assay (A55861 Thermo Fisher Scientific, Waltham, MA, USA). Each sample was plated, containing 500 µg of protein, and incubations were performed for 30 min according to the manufacturers protocol for Group I Mouse Cytokines. Data was acquired on a Luminex 200 plate reader and presented as net mean fluorescent intensity (MFI).

### 2.5. Statistical Analysis

A one-way independent ANOVA with a priori Bonferroni correction was used to assess differences in treatment groups for tumor volume changes flow cytometry populations. Log-rank tests were performed to determine differences in overall survival curves compared to the control for each model (GraphPad Prism Dotmatics, Boston, MA, USA). *p* values < 0.05 were considered significant for log-rank tests. *p* values < 0.01 were considered significant, and *p* values < 0.10 were deemed trending toward significant.

## 3. Results

### 3.1. Low Doses of SAHA Do Not Affect Cancer Cell Viability

**Figure 3:**
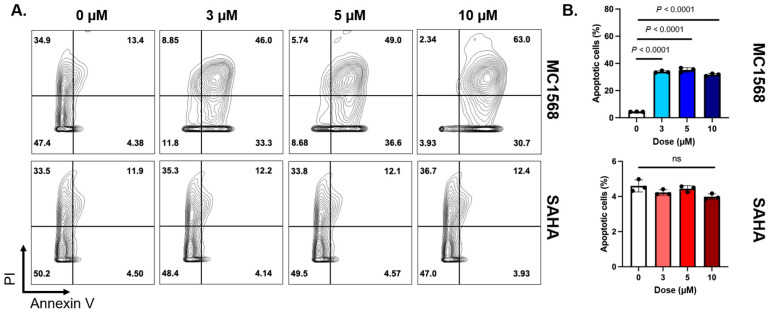
The pan-HDAC inhibitor SAHA does not affect TNBC cell viability. (**A**) Representative plots showing propidium iodide and annexin V staining in 4T1 cells treated with 0, 3, 5, and 10 µM of class II HDAC inhibitor MC1568 and pan-HDAC inhibitor SAHA, respectively (N = 3). (**B**) Quantification of apoptotic (Annexin V^+^PI^−^) cells for each concentration of MC1568 and SAHA.

HDAC inhibitor treatment influences chromatin stability to alter gene expression and cell differentiation, and therefore may impact cell viability. Flow cytometry with PI and annexin V staining was performed to quantify apoptosis and cell viability in 4T1 cells following a 24 h treatment with increasing concentrations of SAHA, a class I and II HDAC inhibitor, or MC1568, a class II HDAC inhibitor. (Figure 3A). We observed that MC1568 robustly promoted apoptosis and cell death, whereas treatment with SAHA showed no discernable effect on apoptosis or necrosis in 4T1 cells (Figure 3B). This data suggests that SAHA treatment alone does not induce significant apoptosis on 4T1 cells in vitro.

### 3.2. Low Doses of SAHA Do Not Affect Cancer Cell Viability Alone

**Figure 4:**
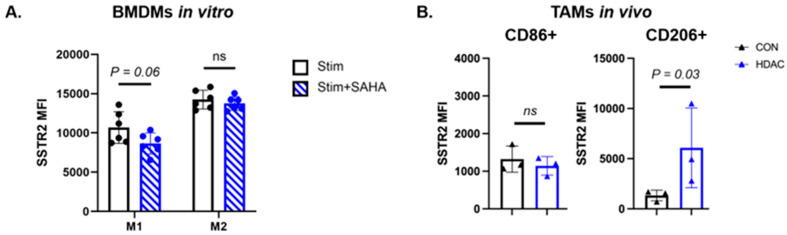
SAHA decreases SSTR2 expression on M1-like BMDMs, but promotes expression on M2-like macrophages in vivo. (**A**) Flow cytometry of bone marrow-derived macrophages (BMDMs) stimulated to the M1-like or M2-like phenotypes treated with SAHA show that SSTR2 expression is decreased on M1-like macrophages, but experienced no change in M2-like macrophages (N = 6 per group). (**B**) Flow cytometry of tumor-associated macrophages (TAMs) show that SSTR2 expression is not changed on CD86+ M1-like macrophages, but increased on CD206+ M2-like macrophages (N = 3 biological replicates per group from separate animals).

Tumor-associated macrophages (TAMs) are an important component of the TME and in some cases express SSTR2, which has been associated with promoting inflammation. To test how HDAC inhibitor treatment influences polarization of macrophages and their expression of SSTR2, we cultured bone marrow-derived macrophages (BMDMs) from BALB/c mice with lipopolysaccharide to induce the M1-like phenotype and IL-4 and IL-13 to induce M2-like phenotypes. We then treated these stimulated BMDMs with vehicle control or 5 µM SAHA for eight hours and performed flow cytometry to quantify SSTR2 expression. We observed that SAHA treatment directly reduces SSTR2 expression on M1-stimulated BMDMs (*p* = 0.06, Figure 4A) compared to control but had no effect on M2-stimulated BMDMs (*p* = 0.45, Figure 4A).

To test whether HDAC inhibitor treatment increases SSTR2 expression on TAMs, we implanted 4T1 cells into the mammary fat pad, waited 12 days for sufficient tumor growth, and began treatment with saline as a control or SAHA every 3 days. On day 7 of treatment, tumors were dissociated and homogenized, and TAMs were analyzed for SSTR2 expression by flow cytometry including CD86+ M1-like TAMs and CD206+ M2-like TAMs. We observed that M1-like 4T1 TAMs had no difference in SSTR2 expression following SAHA treatment (*p* = 0.70, Figure 4B), suggesting that interactions within the TME may be dampening SSTR2 expression, or more likely that other chemokines are responsible for inflammation on day 7 of treatment. M2-like TAMs had significantly increased expression of SSTR2 (*p* = 0.03, Figure 4B). This data supports the hypothesis that the TME plays a critical role in driving pro-inflammatory SSTR2 expression in TAMs beyond epigenetic changes alone.

### 3.3. SAHA Alone and SAHA in Combination with IMT Promotes M1-like Macrophage Recruitment

**Figure 5:**
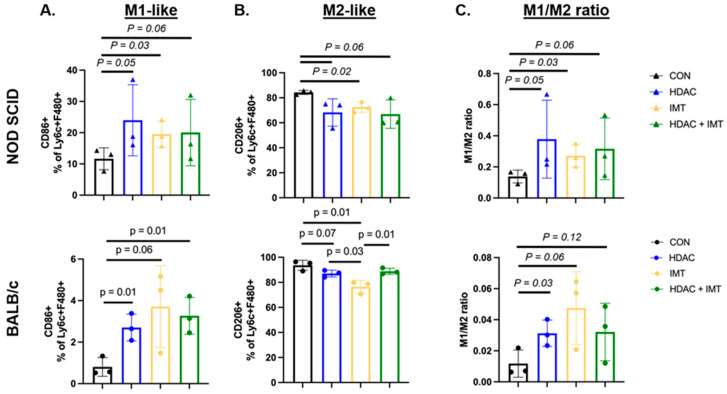
SAHA alone and in combination with IMT promotes recruitment of M1-like macrophages and decreases M2-like macrophages. Populations of CD86+ (M1-like) and CD206+ (M2-like) macrophages are gated on VIA > CD45 + CD11b + >LY6C + F4/80+. One-way independent ANOVA shows significant differences in NOD SCID animals (**top row**, N = 3 biological replicates from independent animals per treatment) between groups for (**A**) M1-like macrophages F(3,8) = 4.15 *p* = 0.04 and (**B**) M2-like macrophages F(3,8) = 29.36 *p* = 0.05, and trending differences in (**C**) M1:M2 ratio F(3,8) = 5.62 *p* = 0.11. One-way independent ANOVA shows significant differences in BALB/c animals (**bottom row**, N = 3 biological replicates from independent animals per treatment) between groups for (**A**) M1-like macrophages F(3,8) = 4.15 *p* = 0.04 and (**B**) M2-like macrophages F(3,8) = 10.45 *p* = 0.003, and trends toward significance for (**C**) M1:M2 ratio F(3,8) = 2.48 *p* = 0.13.

Our data show that single-agent SAHA increases SSTR2 expression on M2-like TAMs. We hypothesized that increased pro-inflammatory SSTR2 expression on TAMs may enhance immune infiltration and activation when combined with checkpoint blockade IMT (α-PD-1 and α-CTLA-4), given their pro-inflammatory effects. To test whether combination SAHA + IMT treatment promotes immune cell infiltration and how adaptive immune cells impact macrophage infiltration, we inoculated immunocompromised NOD SCID mice (lack functional T and B cells) and immunocompetent BALB/c mice with 4T1 mammary fat pad tumors, waited 12 days to establish tumors, and began treatment with saline, single-agent SAHA or IMT, or a combination of SAHA + IMT given every 3 days (N = 3 per group). On day 7 of treatment, animals were euthanized, and tumors were isolated, dissociated, and homogenized for flow cytometric evaluation of TAMs. We observed that SAHA promotes the recruitment of M1-like TAMs alone and in combination with IMT, with additive effects in the presence of adaptive immune cells. SAHA alone and SAHA + IMT increases both M1-like (Figure 5A) Ly6c + F4/80+ recruited macrophages in 4T1 tumors in both NOD SCID and BALB/c animals. Specifically, SAHA drives recruitment of CD86+ M1-like macrophages, which is seen when using it as a single-agent therapy and in combination with IMT.

These results suggest that in the absence of adaptive immune cells, combination therapy promotes anti-tumor macrophage populations and illicit SSTR2 expression on TAMs as a potential driver of inflammation. In the NOD SCID and BALB/c models, we observed decreases in CD206+ M2-like Ly6C + F4/80+ recruited macrophages for all treatment groups compared to control (Figure 5B). We used the ratio of M1:M2 macrophages to determine the overall landscape of tumor-specific macrophages and showed that SAHA increases the M1:M2 ratio in immunocompromised mice, but not as starkly in the immunocompetent model (Figure 5C). This suggests that HDAC inhibition drives macrophage polarization to M1-like, but these macrophages are also influenced by the presence of the adaptive immune populations. This data shows that combination SAHA + IMT drives immune infiltration, specifically, increasing F4/80+ mature macrophage infiltration, and suggests that this recruitment is likely mediated by adaptive immune cells in the TNBC TME, which has been proposed by other groups [26].

### 3.4. SAHA Alone and in Combination with IMT Significantly Increases Overall Survival

**Figure 6:**
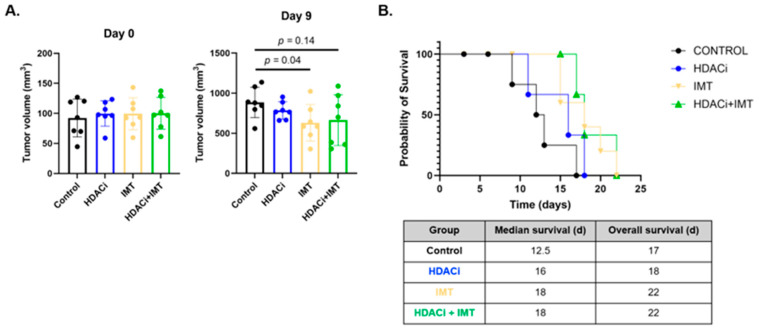
SAHA drives pro-inflammatory macrophages but requires adaptive immunity to decrease tumor burden. (**A**) Overall survival (OS) comparing control, single-agent treatments, and combination SAHA + IMT in 4T1-tumor-bearing BALB/c mice (N = 7 per group). (**B**) OS for single-agent IMT alone (median = 18 d) and combination SAHA + IMT (median = 18 d) is significantly increased compared to controls (median = 12.5 d).

SAHA as a single agent and in combination with IMT promotes anti-tumor immune recruitment and increases pro-inflammatory SSTR2 expression on TAMs at the molecular level, but how these changes in the TME relate to long-term tumor control remain unexplored. Longitudinal survival was used to determine whether combination SAHA + IMT improved outcomes in BALB/c tumor-bearing mice compared to control or single-agent treatments (Figure 6A). Median survival for mice bearing 4T1 tumors is increased for all treatments from 12.5 days for the controls to 16 days for SAHA alone (Figure 6B, *p* = 0.07), and significantly increased to 18 days (Figure 6B, *p* = 0.035) for both IMT alone and combination SAHA + IMT. IMT alone and in combination with SAHA significantly improves longitudinal survival in 4T1-TNBC-tumor-bearing animals. However, while trending towards having an additive effect on promoting anti-tumor macrophages, the combination did not demonstrate increased effectiveness in improving the long-term response compared to IMT alone.

### 3.5. Combination SAHA and IMT Drives Adaptive Immune Response via Chemokine and Cytokine Secretion

Due to the complexities of tumor-immune cell interactions and signaling cascades, we utilized supernatants extracted from homogenized 4T1 tumors to further explore the interactions ongoing within the TNBC TME. We used a multi-plex cytokine assay to characterize how SAHA + IMT treatment influences inflammatory cytokine and chemokine signaling within the tumor-immune landscape, which underlie the effectiveness of the combination in immunocompetent models.

**Figure 7:**
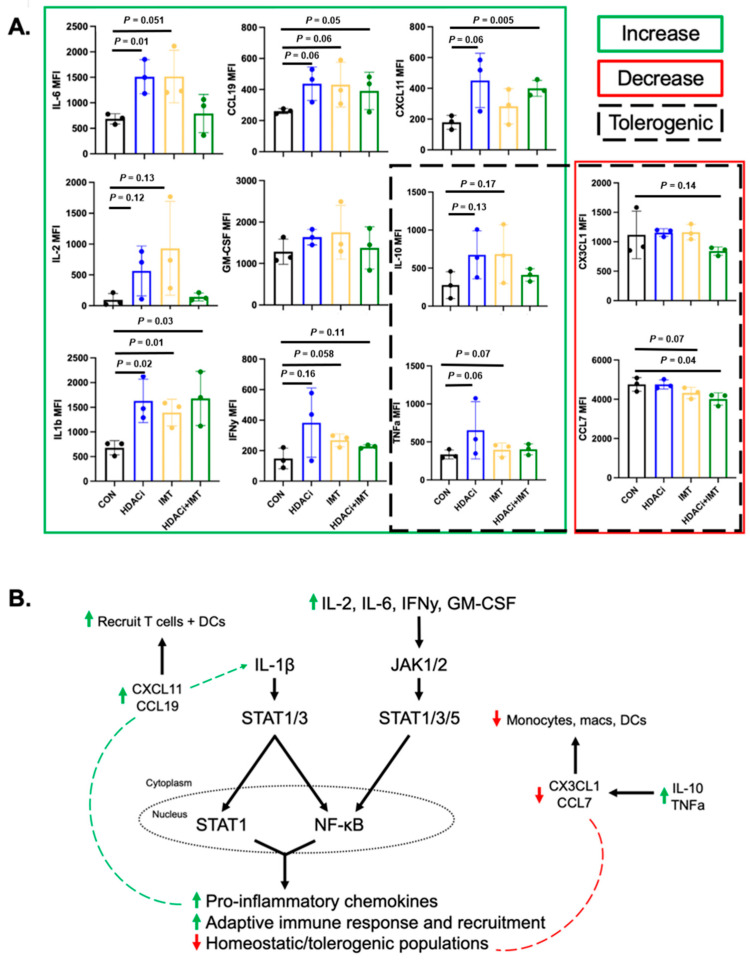
Combination SAHA and IMT drives adaptive immune response while decreasing homeostatic and tolerogenic immune populations via chemokine and cytokine secretion. Cytokines and chemokines collected during tumor processing were evaluated for expression of 31-plex analytes. (**A**) Analysis of chemokines and cytokines important for the recruitment of adaptive immune cells, inflammation, and homeostasis following single-agent or combination treatment including IL-6, IL-2, IL-1β, CCL19, GM-CSF, IFNγ, CXCL11, IL-10, TNFα, CX3CL1, and CCL7. Boxes surrounding chemokines and cytokines represent treatment-induced changes in expression where Green, solid = increase; Red, solid = decrease; Black, dashed = tolerogenic function. (**B**) Proposed mechanism of action behind combination SAHA and IMT driving response in TNBC tumors through canonical NF-κB signaling, which promotes a shift to T cell-mediated response by driving pro-inflammatory chemokines, which recruit T cells, and decreasing tolerogenic immune populations.

We observed trending changes following SAHA and SAHA + IMT treatment in the expression of IL-2 (*p* = 0.12, *p* = 0.13) and IFN-γ (*p* = 0.16, *p* = 0.11), and significant changes in IL-1β (*p* = 0.02, *p* = 0.03, Figure 7A). These cytokines are known promoters of T cell proliferation, differentiation, and activation [17,18]. We also observed significant increased expression of pro-inflammatory IL-6 (*p* = 0.01, *p* = 0.05) and increased, yet not significant, changes in GM-CSF (*p* = ns) following combination treatment, which are important for the recruitment of monocytes and macrophages, in addition to further stimulating inflammatory and wound-healing responses (Figure 7A). IL-6 and GM-CSF have been directly shown to promote tumor progression through the activation of STAT3 and the JAK/STAT pathway, which are primarily secreted by fibroblasts and CD206+ M2-like macrophages [27]. However, our data shows a significant reduction in CD206+ macrophages, suggesting that IL-6 secretion may be coming from tumor cells themselves or from dendritic cells [28]. CXCL11 (*p* = 0.06, *p* = 0.005) and CCL19 (*p* = 0.06, *p* = 0.05), chemokines which promote the recruitment of T cells and dendritic cells, are also increased following SAHA and combination SAHA + IMT treatment. Importantly, we observed less expression of CCL7 (*p* = 0.07, *p* = 0.04) and trending decreases in CX3CL1 (*p* = 0.14), which are potent drivers of macrophage recruitment and accumulation [29,30]. We also observed higher IL-10 (*p* = 0.13, *p* = 0.17) and TNF-α (*p* = 0.06, Figure 7A), yet not significant, which are key pro-inflammatory chemokines that regulate macrophage recruitment and polarization, suggesting a suppression of tolerogenic macrophages.

Overall, cytokine and chemokine data suggest that SAHA + IMT drives inflammatory cytokine secretion, which can recruit anti-tumor immune populations like T cells and dendritic cells and decrease tolerogenic immune populations such as M2-like macrophages. Taken together, we hypothesize that the secretion of these chemokines acts as a positive feedback mechanism for IL-1β signaling (Figure 7B), as a master regulator of inflammation and the T cell-mediated response in TNBC tumors to SAHA + IMT.

## 4. Discussion

Studies have previously demonstrated that treatment with the HDAC inhibitor SAHA can have an anti-tumor function and prime the response to IMT treatment [12,13,31]. While our study showed some moderate increases in survival for SAHA alone, we showed that SAHA in combination with IMT did not have any additional survival benefit compared to IMT alone in a model that responds poorly to IMT. Immunotherapy is typically given to TNBC patients with metastatic or recurrent breast cancer, but it has been expanded to stage I–III disease as well. Despite no additive benefits in survival, this study demonstrates that HDAC inhibitors play a role in macrophage recruitment and influence their expression of SSTR2, which impacts overall treatment outcomes in combination with IMT. Therefore, we anticipate that this specific combination could be useful in scenarios where other therapies, such as chemotherapy or radiation, are not appropriate or the tumors have developed resistance; HDAC inhibitors alone can have direct and indirect effects on the apoptosis of cancer cells through regulation of cell signaling cascades and stimulating death-inducing cells and the secretion of cytokines related to cell death. Our results corroborate these studies by showing that SAHA has drastically different effects in the immunocompromised (NOD SCID) and immunocompetent (BALB/c) models. Our data show that SAHA alone increases M1-like macrophage recruitment and decreases M2-like macrophages in the TME, but these results are amplified in the immunocompetent model, suggesting a more complex interaction with adaptive immune populations and other stromal cells within the TME. Specifically, we believe that dendritic cells, tumor cells, and other stromal cell populations aside from macrophages may be linked to differences in response. IL-6 and GM-CSF have been directly shown to promote tumor progression through the activation of STAT3 and the JAK/STAT pathway, which are primarily secreted by fibroblasts and CD206+ M2-like macrophages [27]. However, our data show a significant reduction in CD206+ macrophages, suggesting that IL-6 secretion may be coming from tumor cells themselves or from dendritic cells [28]. Additional studies exploring adaptive and stromal cell cytokine secretion and regulation of TAMs are warranted to improve our understanding of these relationships in the future. Our cytokine data further support these findings and suggest that SAHA + IMT enhances recruitment of anti-tumor populations; reduces pro-tumoral macrophages though CCL7 and CX3CL1; and promotes a shift toward a pro-inflammatory, anti-tumor immune microenvironment driven by IL-1β signaling.

Alterations in SSTR2 expression have the potential to stimulate strong anti-tumor and pro-inflammatory signaling cascades. The role of SSTR2 expression on macrophages and influence on their function has not been characterized within the breast cancer TME. A limited number of studies have shown that macrophages express SSTR2, but this is the first to investigate SSTR2 expression on TAMs in breast cancer [32]. SSTR2 is expressed on some immune cell populations, and somatostatin receptors have been shown to stimulate immune responses [33,34,35,36]. This work supports the current literature, which suggests SSTR2 expression is variable on macrophages, specifically, on M1-like macrophages, to promote adaptive immune stimulation [36,37]. However, our study newly identifies expression of SSTR2 on M2-like TAMs in the TNBC TME and demonstrates upregulation on these cells following SAHA treatment. As M2-like macrophages have been shown to be tumor promoting, these findings have the potential to help guide combination treatments. SSTR2 expression on tumor-promoting M2-like macrophages provides some evidence why there was no enhanced effectiveness combined with immunotherapy in our model with the known lack of response to immunotherapy. We have demonstrated that SAHA alone and in combination with IMT plays a role in macrophage polarization, recruitment, and SSTR2 expression. We have also identified that SAHA treatment has differing effects on SSTR2 expression on M1-like and M2-like macrophages outside and within the TME. Our data show that SAHA decreases SSTR2 on bone marrow-derived M1-like macrophages, but within the TME, promotes SSTR2 expression on M2-like tumor-associated macrophages. This work demonstrates the pro-inflammatory effects of SAHA + IMT within the TME and the promotion of anti-tumor macrophages, and it explores SSTR2 within macrophage populations and the survival benefit of this therapy. Overall, our studies demonstrate the ability of SAHA to modulate SSTR2 expression in macrophages and demonstrate the importance of the tumor microenvironment in SSTR2 expression and overall treatment response outcomes. Identifying cellular and therapeutic effects of novel combination therapies in TNBC can inform treatment strategies and potentially identify additional biomarkers for imaging and therapy, such as CD206+ macrophages or SSTR2 expression.

An important consideration for SSTR2 expression on macrophages within the TME is in the context of imaging and theranostic approaches targeting SSTR2. SSTR2-targeted theranostic agents are FDA approved for use in neuroendocrine tumors and are currently being investigated for other indications and cancer types, including hepatocellular carcinoma (NCT03648073), glioblastoma (NCT05109728), meningioma (NCT03971461 and NCT04082520), neuroblastoma (NCT05109728 and NCT03966651), thyroid cancer (NCT04927416), and advanced or recurrent breast cancer (NCT04529044). Previous studies from our lab have demonstrated the ability of SAHA to upregulate SSTR2 expression on tumor cells to create a target for imaging and potentially theranostics in preclinical TNBC models [22]. In this study, we have demonstrated that SSTR2 expression is upregulated on M2-like macrophages in the TNBC TME following SAHA treatment. This data suggests that theranostic delivery of targeted radiotherapy could target not only tumor cells but also reduce pro-tumoral M2-like macrophages. This could provide a two-pronged approach to increase tumor killing. As theranostics are expanded, it will be critical to quantify the relationship of SSTR2 on tumor and macrophage populations to understand how both populations are affected by both HDAC inhibition alone and in combination with targeted radiotherapy.

Future studies are needed to explore the effects of HDAC and immunotherapy in combination at the transcriptional level to provide better understanding of the downstream, cellular changes within the TNBC TME. Additional dynamic evaluation of these therapies at various time points during therapy will be necessary to further understand the effects of combination SAHA + IMT on adaptive immune cells and innate populations outside of macrophages. Specifically, characterizing T cells, natural killer cells, and dendritic cells will be of interest given their role in mediating antigen presentation and tumor cell killing following HDAC inhibitor treatment and checkpoint blockade IMT [9,38]. In addition, our flow cytometry and cytokine data have strongly indicated a role for adaptive immunity in mediating tumor response, which has been well established for checkpoint blockade IMT. Our data have demonstrated that HDAC inhibitors drive innate immunity in TNBC, but ultimately, these populations are not sufficient to enhance survival outcomes compared to single agents. 4T1 mammary carcinoma is known to be a fast-growing, aggressive model with limited response to immunotherapy and the high presence of TAMs (30–70), which is a potential limitation of this work; however, we do acknowledge there is wide clinical variation of TNBC growth patterns. We believe exploring SAHA + IMT in a more immunogenic mouse background, C57BL6 mice with syngeneic E0771 tumors, could be useful to understand these interactions, specifically given the disposition to M1-like macrophage predominance in this model. Exploring this design in breast cancer models with an increased percentage of response to immunotherapy, or clinically relevant patient-derived xenograft models, may provide additional key evidence to understand how to expand combination treatments for translation. Further understanding how combining HDACi with IMT influences other immune populations is crucial, as these therapies are currently being investigated in clinical trials (HDACi) and standard-of-care (IMT) for TNBC and may be combined in the future for theranostics.

## 5. Conclusions

Overall, our study shows that SAHA modulates the TNBC TME by altering macrophage recruitment, polarization, and expression of SSTR2. SAHA alone and in combination with IMT promotes recruitment of M1-like macrophages, while reducing M2-like macrophages. Additionally, these changes in macrophages are reflected in cytokine and chemokine analysis, which reveals an increase in inflammatory markers and reducing tolerogenic and immunosuppressive markers. IMT alone and in combination with SAHA significantly improved overall survival, with the combination having additive effects at promoting an anti-tumor signaling cascade. One key finding from our study is the differential effect of SAHA and the TME on SSTR2 expression. SAHA reduces SSTR2 on M1-like macrophages in vitro, but upregulates SSTR2 on M2-like TAMs in vivo. This novel observation suggests a role for SSTR2 in promoting immune responses, as previously suggested by others (references), and has important implications in SSTR2-targeted theranostic approaches. Given the limited survival benefit of SAHA alone, but robust enhancement of anti-tumor populations and cytokines, combining SAHA with IMT and potentially theranostic agents could offer stronger immune-driven anti-tumor effects in TNBC.

## Data Availability

The original contributions presented in this study are included in the article/Supplementary Material. Further inquiries can be directed to the corresponding authors.

## Supplementary Materials

The following supporting information can be downloaded at: https://www.mdpi.com/article/10.3390/pharmaceutics18050539/s1, Figure S1: Apoptosis flow cytometry gating strategy; Figure S2: Macrophage flow cytometry gating strategy; Table S1: Flow cytometry conjugates used for cell staining and assessment of apoptosis (AF488-Annexin V and Propidium iodide) including target binding, dilutions, and catalog information.

## Abbreviations

The following abbreviations are used in this manuscript:

TNBC	Triple-negative breast cancer
HDAC	Histone deacetylase
IMT	Immunotherapy
SSTR2	Somatostatin receptor subtype 2
SAHA	Suberoylanilide hydroxamic acid
BMDM	Bone marrow-derived macrophage
TME	Tumor microenvironment
TAM	Tumor-associated macrophage
NOD SCID	Non-obese diabetic/severe combined immunodeficiency
FACS	Fluorescence-associated cell sorting
PI	Propidium iodide
CD#	Cluster of differentiation
CCL#	C-C motif chemokine ligand
CXCL#	C-X-C motif chemokine ligand
IL-#	Interleukin
IFN-γ	Interferon gamma
GM-CSF	Granulocyte-macrophage colony-stimulating factor
TNF-α	Tumor necrosis factor alpha
MFI	Mean fluorescent intensity
