# Supplementary material for: SAHA Alters Macrophages in the Tumor-Immune Landscape in Preclinical Models of Triple-Negative Breast Cancer
Source: Pharmaceutics. 2026 Apr 28;18(5):539. doi: 10.3390/pharmaceutics18050539 (PMC13210165; doi:10.3390/pharmaceutics18050539)
Supplement: Supplementary file 1 [file pharmaceutics-18-00539-s001.zip › pharmaceutics-4235120-supplementary.pdf]

Supplementary Data

Table S1. Flow cytometry conjugates used for cell surface staining and assessment of apoptosis (AF488-Annexin V and Propidium iodide) including target binding, dilutions, and catalog information.

Fluorophore-conjugates	Target	Dilution	Catalog number
FITC-SSTR2	Cell surface	1:32	Novus, 402038
PerCP-Cy710-Arginase 1	Intracellular	1:64	eBioscience, 46-3697-82
APC-MHC Class II I-A/I-E	Cell surface	1:100	Invitrogen, 17-5321-81
AF700-IFNy	Intracellular	1:32	eBioscience, 56-7319-42
NIR-Live/Dead	Cell surface and intracellular	1:150	ThermoFisher, L34975
eFluor450-Ly6C	Cell surface	1:64	ThermoFisher, 48-5932-82
BV510-CD45	Cell surface	1:64	BD Biosciences, 563891
SB645-F4/80	Cell surface	1:100	ThermoFisher, 64-4801-82
SB702-CD86	Cell surface	1:32	eBioscience, 67-0862-82
SB780-CD11b	Cell surface	1:64	ThermoFisher, 78-0112-82
PE-eFluor610-CD206	Cell surface	1:32	ThermoFisher, 61-2061-82
AF488-Annexin V (Apoptosis)	Cell membrane, Phosphatidylserine (PS)	1:100	ThermoFisher, A13201
Propidium iodide (Apoptosis)	Intracellular, DNA	1:50	ThermoFisher, P1304MP

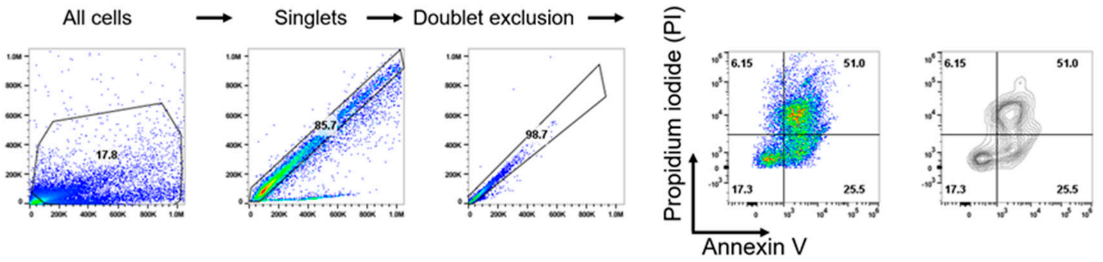

Figure S1. Apoptosis flow cytometry gating strategy. A. Flow cytometry gating strategy for in vitro assessment of apoptosis.

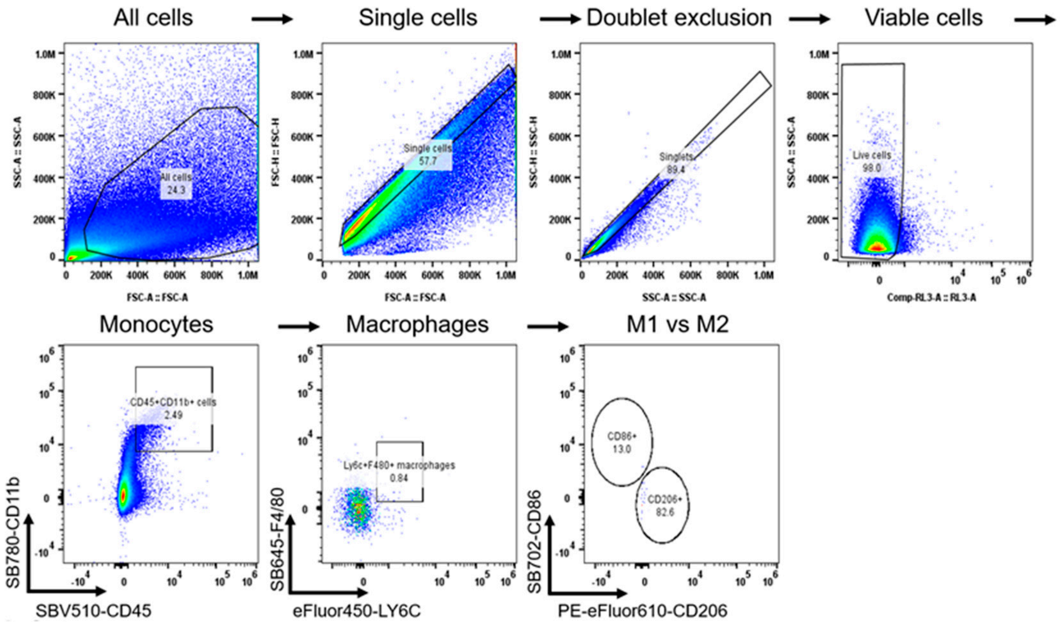

Figure S2. Macrophage flow cytometry gating strategy. A. Flow cytometry gating strategy for in vivo assessment of tumor-associated macrophages (TAMs).
